# Factors Affecting Seed Germination of the Invasive Species *Symphyotrichum lanceolatum* and Their Implication for Invasion Success

**DOI:** 10.3390/plants11070969

**Published:** 2022-04-01

**Authors:** Marija Nešić, Dragica Obratov-Petković, Dragana Skočajić, Ivana Bjedov, Nevena Čule

**Affiliations:** 1 Department of Landscape Architecture and Horticulture, Faculty of Forestry, University of Belgrade, Kneza Višeslava 1, 11030 Belgrade, Serbia; dragica.obratov-petkovic@sfb.bg.ac.rs (D.O.-P.); dragana.skocajic@sfb.bg.ac.rs (D.S.); ivana.bjedov@sfb.bg.ac.rs (I.B.); 2 Department of Environmental Protection and Improvement, Institute of Forestry, Kneza Višeslava 3, 11030 Belgrade, Serbia; nevena.cule@yahoo.com

**Keywords:** *Symphyotrichum lanceolatum*, invasive plants, generative reproduction, seed, invasion success, disturbed sites

## Abstract

Invasive species *Symphyotrichum lanceolatum* (Willd.) G. L. Nesom is spreading uncontrollably along wet habitats as well as in disturbed ecosystems. All those habitats function as corridors that facilitate seed dispersal. One way to prevent the spread of invasive species is to know their reproductive ecology. The present study evaluates the potential for generative reproduction of *S. lanceolatum* and determines how different temperatures, amounts of nutrients, and light regimes, affect seed germination. Seeds collected from 13 natural populations were germinated at four fluctuating temperature regimes (15/6, 20/10, 30/15, and 35/20 °C). To test the influence of nitrate on seed germination, two KNO_3_ concentrations were used (5 mM and 50 mM solution). For each treatment, three replicates of 30 seeds were placed in complete darkness or a 14 h photoperiod. The results showed that the germination increased with increasing temperature. The optimal temperature regimes were 30/15 °C and 35/20 °C with approximately 88% germination. The overall effect of KNO_3_ on germination was positive. The concentration of 50 mM KNO_3_ had a less stimulating effect compared to 5 mM KNO_3_. Seeds showed sensitivity to lack of light during germination but were able to germinate in a significant percentage in such conditions. Considering that *S. lanceolatum* often occurs in disturbed sites, these results suggest that seed reaction to alternating temperature, nutrients concentration, and light can be determining factors that affect seed germination of this species and, thus, its spread.

## 1. Introduction

Invasive species are one of the biggest threats to world biodiversity, contributing to landscape homogenization by forming large but species-poor stands [1]. By the most frequently used definition, invasive plants have the potential to spread over large areas by producing reproductive offspring, often in large numbers and at considerable distances from the parent plant [2].

When an introduced species enters a new habitat outside its previous geographical range, it faces new climatic and habitat conditions. This poses a challenge for introduced species to establish a population that will further spread to new habitats [3]. Moravcova et al. [4] found that reproductive traits, especially those related to seed production and dispersal, have the greatest impact on the assessment of introduced plants invasiveness.

The size and number of seeds produced by invasive plants affect the species’ ability to colonize new habitats and to form a stable population [5]. The literature on invasive species traits has highlighted that many invasive species produce large quantities of small seeds [6,7,8,9]. *Symphyotrichum lanceolatum* (Willd.) G. L. Nesom (Compositae) is one of the plant species that produce very large numbers of small seeds. Small seeds usually remain viable longer in the soil and form more permanent seed banks [4,10]. Equally important is that plants with the ability to quickly colonize disturbed habitats have small seeds that are dispersed over long distances. Hence, species with such seeds are more likely to become invasive [4,8,11].

Propagule pressure is a term that describes the number or rate at which propagules enter a particular area [12]. However, the quality of propagules should be considered when assessing the propagule pressure or the probability of species invasion [13]. Propagule pressure is a factor that plays a crucial role in the colonization of new habitats [14,15].

The spread of introduced species continues due to adaptions for seed dispersal [16]. Generative reproduction allows propagule dispersal over long distances. In contrast, vegetative propagules rarely possess mechanisms that allow them to be dispersed over long distances [17,18]. Nevertheless, it is also important to note that there are many cases of introduction of clones or vegetative propagules (bulbs, bulbils) that are at the base of many cases of invasion [19,20,21].

The seed is sensitive to changes in environmental conditions such as temperature, light, moisture, and nitrate content [22]. Nitrate can regulate germination both alone or in combination with other abiotic factors, such as alternating temperature or light [22,23]. Increased nitrogen availability in soil could help break seed dormancy and stimulate germination [24]. Furthermore, an increase in the amount of nitrogen in the soil due to the deposition of atmospheric nitrogen can increase the dominance of invasive plant species [25]. The seed reaction to light is also the mechanism by which the seed responds to the sudden disappearance of vegetation after detecting a high concentration of nutrients, and thus the lack of competition [26]. The positive reaction of the seed to alternating temperatures and the increased nitrate content is also the mechanism by which the seed reacts to the vegetation gaps. Plants that occupy clearings and have strong growth have an advantage over plants that will appear in the later stages of succession when the competition for resources is higher [27].

The joint effects of several environmental factors are implied in natural habitats [26]. Even though such interactions complicate the interpretation of the experimental results, they provide an assumption of species functioning under natural conditions. Vincent and Roberts [28] found that the combination of cold stratification, nitrate, alternating temperature, and different light regimes has several positive effects on the seeds of many weed species [29].

*S. lanceolatum* is a tall, herbaceous perennial occupying early successional habitats. The species is native to North America and Canada, and invasive in many European countries [30]. *S. lanceolatum* blooms and bears fruits in late summer to late autumn and aboveground parts die back subsequently [31,32]. Achenes mature 3 to 4 weeks after pollination, and new seedlings occur in spring [31]. This species inhabits moist, nitrogen-rich soil along riverbeds and lakes, ponds, roads, as well as wet ecosystems in which disturbances are expressed [33,34,35,36]. All these habitats act as corridors through which plants spread.

The balance between generative and vegetative reproduction, seed size, dormancy, seed dispersal time and mechanisms, and life span is the main reproductive characteristic of wetland plants [37]. Accordingly, we anticipated that traits that favor *S. lanceolatum* over native species and allow this species to quickly colonize new habitats are related to its reproductive potential. Therefore, the present study evaluates the generative reproduction of *S. lanceolatum* and determines how different light regimes, temperatures, and amounts of nutrients affect seed germination. Considering that *S. lanceolatum* occupies habitats in which disturbances are expressed, it is expected that alternating temperatures and increased nutrients will have a positive effect on seed germination.

## 2. Results

### 2.1. Generalized Effect of Alternating Temperatures, Nitrates, and Light on Seed Germination

Three-way ANOVA was used to test the effects of temperature, KNO_3_ concentration, and light condition on germinative capacity (GC). Looking at Table 1, it is apparent that GC was significantly affected by temperature, light, and KNO_3_ concentration, as well as by interaction between temperature and light, temperature and KNO_3_ concentrations, and light and KNO_3_ concentrations.

**Figure 1:**
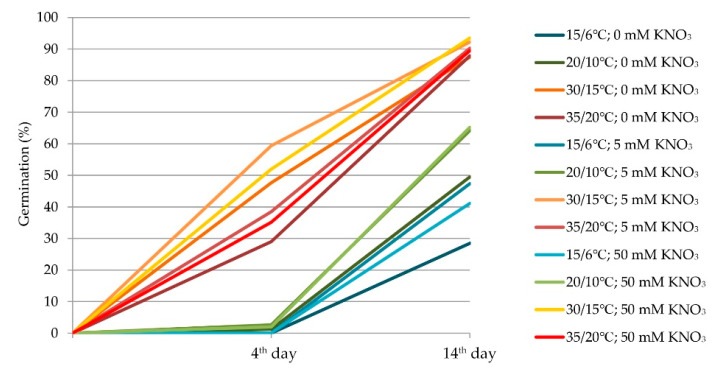
Dynamics of seed germination (%) under light conditions at the different temperature regimes and KNO_3_ concentration.

Figure 1 displays cumulative germination data for all combinations of incubation temperature and nitrate concentration under light conditions. As can be seen, the germination dynamic was similar at 15/6 °C and 20/10 °C on the fourth day and germination was neglectable. However, final germination was higher at 20/10 °C (49.49–65.21%) compared to 15/6 °C (28.46–47.35%). At elevated temperatures (30/15 °C and 35/20 °C), germination was higher compared to other temperature regimes, with germination on the fourth day ranging between 28.7 and 59.4%. After that, a similar germination dynamic can be seen since the percentage of final germinated seeds was around 90% in those elevated temperature regimes.

### 2.2. Effect of Alternating Temperatures on Seed Germination

Table 2 reveals that there was an increase in GC with increasing temperature, at both light regimes in treatments with KNO_3_ and control. In all treatments and both light conditions, GC was the lowest at 15/6 °C. Seeds exposed to light germinated in a higher percentage. Further, GC was higher at higher temperatures (30/15 °C and 35/20 °C) in both light regimes. The exception was treatment with 5 mM KNO_3_ in dark conditions, where GC was significantly lower at 35/20 °C compared to 30/15 °C treatment (Table 2).

### 2.3. Effect of Nitrate on Seed Germination

**Figure 2:**
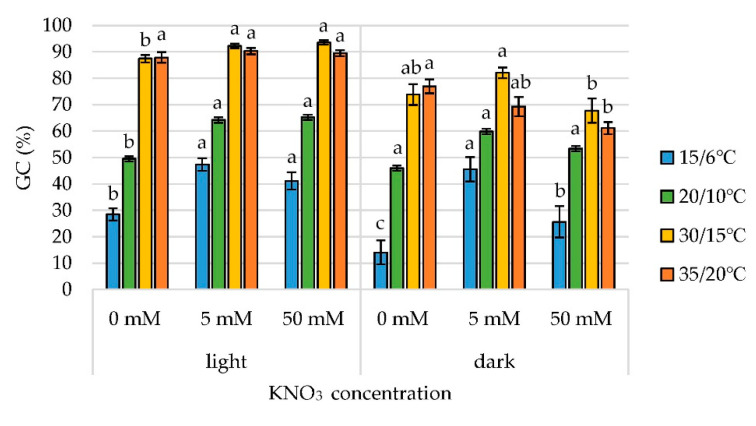
The effect of KNO_3_ concentration, within the same temperature regime, on germinative capacity (GC) in light and dark treatments. Each value represents the mean ± SE for three repetitions. Mean values within light/dark treatment and same temperature with different letters differ significantly, *p* < 0.05.

Figure 2 shows that a significant increase in GC was caused by both KNO_3_ treatments compared to control in light at all temperature regimes except for 35/20 °C, where the increase was not significant. The 5 mM KNO_3_ treatment also had a positive effect on GC increase in the dark, except at a temperature of 35/20 °C, where GC was lower, but without statistical significance (*p* > 0.05). Treatment with 50 mM KNO_3_ increased the GC compared to control (*p* < 0.05) at all temperature regimes, except at 35/20 °C in light conditions (Figure 2). In dark conditions, GC increased only at 15/6 °C. The 50 mM KNO_3_ treatment did not have a statistically significant effect on GC compared to control at 20/10 °C and 30/15 °C in dark. Furthermore, GC was significantly lower at 35/20 °C compared to control.

### 2.4. Effect of Light on Seed Germination

**Figure 3:**
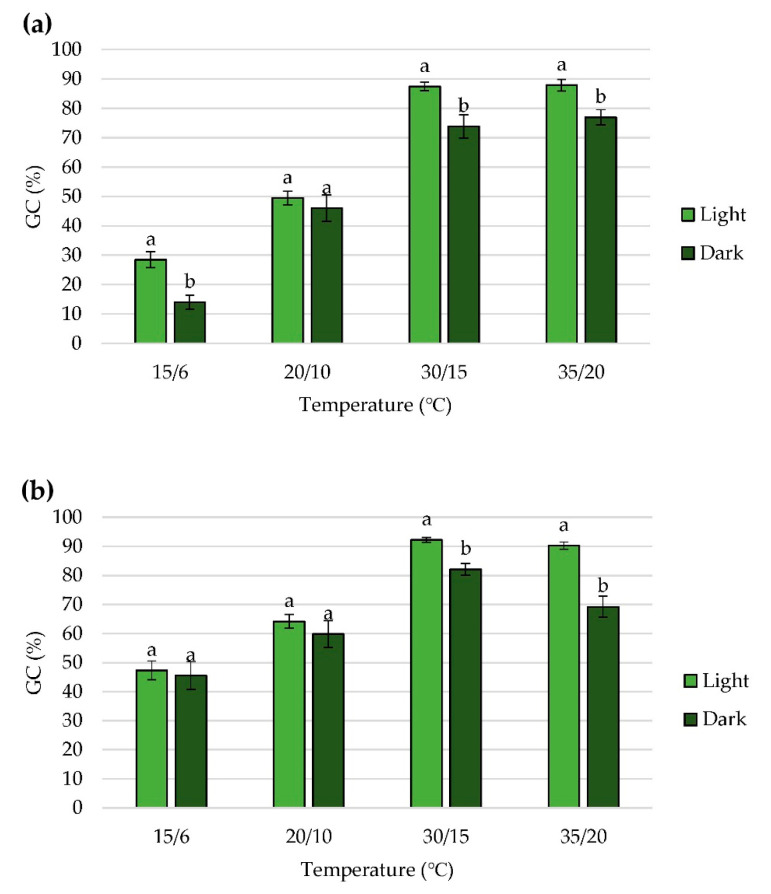

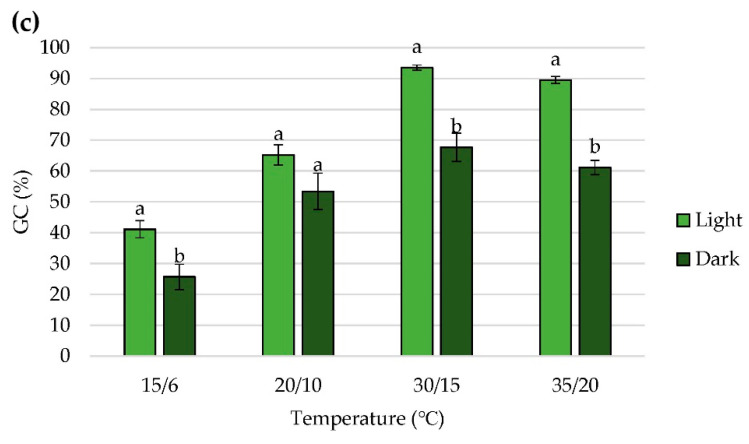
The effect of light, within the same temperature regime, on germinative capacity (GC) in treatments with (**a**) 0 mM KNO_3_; (**b**) 5 mM KNO_3_; (**c**) 50 mM KNO_3_. Each value represents the mean ± SE for three replicates. Mean values within the same temperature treatment with different letters differ significantly, *p* < 0.05.

As expected, seed germination was inhibited in the dark treatments (Table 3). Results showed that GC in the dark was lower than GC in the light (Figure 3). The lack of light significantly reduced the GC by 50.75% in the treatment with 0 M KNO_3_ (control) at 15/6 °C (Table 3, Figure 3a). A statistically significant decrease was also recorded at temperatures 30/15 °C and 35/20 °C in all KNO_3_ treatments (Figure 3a–c). The smallest germination inhibition by dark was in the treatment with 5 mM KNO_3_ at 15/6 °C (Table 3). This result is in line with the results of the ANOVA test (Figure 3b) which showed no significant difference between GC under light and dark conditions. In contrast, in the treatment with 50 mM KNO_3_, a statistically significant inhibition of germination was recorded at 15/6 °C (Figure 3c), pointing to the highest germination inhibition (37.63%) by this treatment (Table 3).

## 3. Discussion

Jones (1978) showed that *S. lanceolatum* germination percentage ranges between 60–80% in seeds that have not been stratified, while after cold stratification this percentage was even higher. However, Schmid and Bazzaz [38] determined that the *S. lanceolatum* germination percentage for seed germinated at a 24 °C day and an 18 °C night temperature cycle ranged between 39.5–43.7%. Our previous study showed that the *S. lanceolatum* germination percentage was below 46% for seeds that germinated at 20 °C [32]. In contrast, the current study showed much higher germination in elevated, alternating temperatures. Germination was also enhanced in treatments with potassium nitrate. Although the results showed a higher percentage of germinated achenes in relation to our initial research, these results differ from the Jedlička and Prach [30] study which suggested very high germination, even 100%, for seeds that were germinated immediately after collection, after storage at room temperature for 5 months or after storage at 5 °C for 5 months, at 25/15 °C day–night temperature regime. Our study has been unable to demonstrate such a high percentage of germination for seeds that have been stored at a temperature between 2.3 and 6.9 °C. Interspecific hybridization between species of *Symphyotrichum* is very common [31]. It may be the case, therefore, that these variations could have contributed to different germination percentages. In the case of hybrids, germination can be as low as 10% [39].

In seed ecology, temperature has a dual role. It affects dormancy as well as seed germination [40,41]. *S. lanceolatum* seeds ripen in late November or early December when their dispersal begins and lasts throughout the winter. Seeds that fall on the soil surface during autumn and winter do not germinate, because the minimum temperature required for germination is higher than the air and soil temperature in that period [42]. Further, according to the same authors, seed stratification during the winter, at low temperatures, reduces the minimum temperature required for germination. This process allows the seeds of genus *Symphyotrichum* to germinate in the spring at a temperature that would be low for seed germination during the fall. In non-dormant seeds, temperature affects the percentage of seed germination [43]. Considering that the seeds used in this experiment underwent a process of after-ripening for 4 months, at a temperature between 2.3 and 6.9 °C which overcame seed dormancy, it can be concluded that the temperature had a significant effect on the germination parameters, which is confirmed with ANOVA tests. This claim is also supported by the results of our previous research, with seeds collected from the same populations [32]. In this research, the seeds were stored at 5 °C for an after-ripening period of 4 months and were germinated at 20 °C as advised by the ISTA standards [44] for species of genus *Symphyotrichum*, while the light regime was 16/8 h. The study showed low seed germination (from 0.58% to 45.98%). In the current research, germinative capacity at 20/10 °C, under light conditions, ranged from 3.33 to 53.33%. However, the reason for higher germination might be the alternating temperature, which in some species has a positive effect on germination parameters [45]. Our results support this claim since GC was significantly higher at elevated temperatures (30/15 °C and 35/20 °C) with germination reaching 88%.

Nitrates affect the loss of dormancy [29,46], and in seeds that are not dormant, they can increase germination [45]. Seed germination under the influence of nitrate is also affected by other environmental conditions, especially by light and alternating temperature [47]. In this study, potassium nitrate showed a stimulating effect on the seeds of *S. lanceolatum.* The concentration of 50 mM KNO_3_ had a less pronounced stimulating effect compared to 5 mM KNO_3_. However, the inhibitory effect of both KNO_3_ concentrations was notable in the dark at the highest temperature. As already mentioned, GC increased with temperature which suggests that germination was under thermal control but was also enhanced with KNO_3_ since germination percentage was even higher when seed germinated in KNO_3_ treatments, in contrast to control. A positive effect of KNO_3_ was especially noticeable at 15/6 °C which suggest that KNO_3_ in both applied concentrations can substitute the requirement of seeds for temperature [42]. Small seeds are often photoblastic, or their germination is significantly inhibited by a lack of light [45,48]. Germination of *Symphyotrichum pilosum* seeds is controlled by phytochrome [49]. Red and white light encourage germination, and darkness and far-red light inhibit it [42,50]. Seeds of *S. lanceolatum* showed less sensitivity to lack of light during germination. Unexpectedly, the smallest decrease in seed germination from the darkness treatment was observed at 15/6 °C in the treatment with 5 mM KNO_3_. In some cases, the inhibitory effect of lack of light can be reduced by the influence of nitrate and alternating temperature [26]. The ability of *S. lanceolatum* seeds to germinate in the dark is a significant ecological characteristic. This means that if the seed happens to be covered with soil or dry plant material before the end of winter, there is a possibility that it will germinate. This is especially important for the *S. lanceolatum* reproductive ecology because the species inhabits habitats where disturbances in ecosystems are pronounced [51,52].

The sudden disappearance of vegetation in one part of the ecosystem can occur as a consequence of a disturbance caused by natural or anthropogenic factors. Competition for resources is low in those gaps, so new plants can take advantage of available resources. Seed germination is the most important step in the growth cycle of a plant [53]. To use available resources, plants have developed physiological mechanisms by which they recognize environmental conditions that correspond to the conditions that arise after the sudden disappearance of vegetation [54,55]. Further, an alternation of temperature can stimulate the small seeds’ germination because it indicates the sudden disappearance of vegetation or shallowly buried seeds [45]. Pons [29] points out that the reaction of seeds to nitrates is also a mechanism by which seeds can respond to the sudden disappearance of vegetation after detecting a high concentration of nutrients. Then, Fenner and Thompson [45] suggest that the substantial proportion of red light indicates a lack of vegetation and thus a lack of competition. According to the same authors, the ability to react to some environmental conditions allows the seed to have some control over when and where it will germinate. The current study found that temperature significantly increased germination percentage, especially at elevated temperatures (30/15 °C and 35/20 °C), as well as KNO_3_ in both concentrations (5 mM and 50 mM). Emphasizing that *S. lanceolatum* occurs in disturbed ecosystems, taken together, the findings of this study suggest that the reaction to changes in temperature, amount of nutrients, and light is one of the decisive factors influencing the germination of seeds of this species. Seeds were able to germinate at different temperature regimes, nutrient concentrations, and different amounts of available light. These results provide further support for the hypothesis that due to seed characteristics and its adaptability, this species can gain an advantage over other indigenous species that coexist in the same habitat.

## 4. Materials and Methods

### 4.1. Experimental Setup

At the initial phase of the investigation, 21 localities were investigated in Serbia. Subsequently, 13 localities where *S. lanceolatum* occurs with high abundance and frequency were chosen for this study. Seeds from standing plants and different parts of inflorescences were collected from those 13 natural populations during the autumn (November 2017) to test the *S. lanceolatum* seed germination. All selected localities were located along the ecological corridors of the Sava and the Danube. Eight sites close to the Sava and Danube rivers were selected in the wider territory of Belgrade. Additionally, five more sites were investigated near the Danube River 75 km north of Belgrade. The climate of the researched area was moderately continental, altitudes ranged from 71 to 200 m, and soil was mainly a haplic fluvisol (calcaric).

Mature achenes from all populations were stored in Belgrade for 4 months (from December 2017 to March 2018) at a temperature between 2.3 °C and 6.9 °C. Achenes were stored in dry conditions (after ripening) by exposing them during the fall and winter months to outdoor temperatures. Average monthly air temperatures were 4.8 °C in December 2017, and 5.3 °C, 2.3 °C, 6.9 °C in January, February and March 2018, respectively [56,57]. Subsequently, the germination test was performed by the direct method in the plant growth chamber SANYO MLR-351H. Seed germination was examined concerning several environmental factors. In addition, seed viability percentage was determined by the tetrazolium test [44].

Seeds were placed in Petri dishes (9 cm) between two layers of filter paper moistened with 10 mL of distilled water to determine the effect of 4 alternating temperature regimes on germination [44]. Germination tests were carried out at 4 alternating temperature regimes: 15/6, 20/10, 30/15, and 35/20 °C [42]. Two KNO_3_ concentrations were used: 5 mM and 50 mM solutions [58,59], on 4 temperature regimes to test the influence of nitrate on seed germination. Distilled water was used for control (0 mM KNO_3_). For each treatment, three replicates of 30 seeds were placed in complete darkness or a 14 h photoperiod, with a photon flux density of 27 μmol m^−2^ s^−1^ which was provided by fifteen, 40 w growth chamber fluorescent lamps. Air humidity was 70%. The effects of treatments applied were quantified as germinative capacity (GC), in both light treatments [44]. GC was calculated after 14 days as the number of seeds that had produced seedlings classified as normal [44], as a proportion (%) of the total number of tested seeds.

### 4.2. Data Analysis

Germination data were transformed to percentages based on the number of germinated seeds, and the viable seeds were calculated as the sum of fresh non-germinated and germinated seeds, also expressed as the mean (%) of the number of seeds in 3 replicates.

Differences in germination means among treatments were analysed by three-way ANOVA. Three-way ANOVA was used to determine the effects of temperature (T), KNO_3_ concentration (K), and light condition (L) on germinative capacity (GC). If an ANOVA indicated significant differences in the data, Fisher’s LSD test was used to determine the differences among treatments (*p* < 0.05). All analyses were performed with Statgraphics Centurion XVI (Statpoint Technologies, Inc., Warrenton, VA, USA).

In treatments with the same temperature regime and the same KNO_3_ concentration, the effect of light was expressed as % of germination inhibition in the dark relative to seed germination in light, based on the formula:

% growth inhibition = 100 (GCd − GCl)/GCd(1)

where GC_d_ and GC_l_ are seed germinative capacity in treatment in darkness and light, respectively.

## 5. Conclusions

The present study was designed to determine the potential for generative reproduction of *S. lanceolatum*. The second aim was to determine how different light regimes, temperatures, and amounts of nutrients affect seed germination. This study has identified that elevated alternating temperature and nutrient concentration increased *S. lanceolatum* germination. GC was significantly higher at elevated temperatures (30/15 °C and 35/20 °C). Furthermore, potassium nitrate had a positive effect on the seed germination, and higher GC was recorded in the treatment with 5 mM KNO_3_ compared to the 50 mM KNO_3_ concentration. Furthermore, *S. lanceolatum* seeds showed sensitivity to lack of light during germination. However, the seeds have also shown the ability to germinate in a significant percentage in such conditions. The findings of this research provide an insight into one of the possible ways that *S. lanceolatum* spreads to new habitats, and that is via seeds. Generative reproduction can play a significant role in this process since *S. lanceolatum* produces a high number of viable seeds which can germinate in a relatively high percentage in different environmental conditions. Considering that *S. lanceolatum* often occurs in disturbed sites, seed reaction to changes in temperature, nutrient concentration, and light is one of the determining factors that affect seed germination of this species and, thus its spread. Further studies regarding the role of generative reproduction would be worthwhile. These studies should explore whether and at what distance from parent plants seedlings of this species occur. In addition, it would be interesting to determine the seedlings’ survival and whether that is enough to establish new populations.

## Figures and Tables

**Table 1:** Analysis of variance of germinative capacity (GC) of *S. lanceolatum* affected by temperature regimes (15/6 °C, 20/10 °C, 30/15 °C, and 35/20 °C), KNO_3_ concentration (0 mM, 5 mM, and 50 mM) and light regimes (14 h light photoperiod and total darkness).

Source of Variation	df	MS	F	*p*
Temperature (T)	3	121,357.95	303.84	<0.001
Light (L)	1	42,315.05	105.94	<0.001
KNO_3_ (K)	2	9324.94	23.35	<0.001
T × L	3	2132.10	5.34	<0.001
T × K	6	3220.27	8.06	<0.001
L × K	2	2847.56	7.13	0.001
T × L × K	6	503.64	1.26	0.272
Error	912	399.41		
Total	935			

df = degrees of freedom; MS = mean square; F = F-ratio.

**Table 2:** The effect of alternating temperature and applied KNO_3_ concentration under the different light conditions on the germinative capacity (GC) of *S. lanceolatum*.

Light Treatment	Temperature (°C)	0 mM KNO_3_	5 mM KNO_3_	50 mM KNO_3_
		Germination Capacity (%)		
Light	15/6	28.46 ± 2.72 ^c^	47.35 ± 3.21 ^c^	41.11 ± 2.78 ^c^
	20/10	49.49 ± 2.31 ^b^	64.19 ± 2.41 ^b^	65.21 ± 3.28 ^b^
	30/15	87.44 ± 1.41 ^a^	92.22 ± 0.85 ^a^	93.50 ± 0.85 ^a^
	35/20	87.86 ± 1.97 ^a^	90.26 ± 1.26 ^a^	89.49 ± 1.17 ^a^
Dark	15/6	14.02 ± 2.39 ^c^	45.56 ± 4.76 ^c^	25.64 ± 4.12 ^c^
	20/10	45.98 ± 4.52 ^b^	59.83 ± 4.60 ^b^	53.33 ± 5.89 ^b^
	30/15	73.85 ± 3.96 ^a^	82.05 ± 2.07 ^a^	67.69 ± 4.59 ^a^
	35/20	76.92 ± 2.58 ^a^	69.23 ± 3.65 ^b^	61.11 ± 2.33 ^ab^

Each value represents the mean ± SE for three replicates. In the same light treatment, mean values with different letters differ significantly, *p* < 0.05.

**Table 3:** Germination inhibition in dark treatments.

Temperature (°C)	0 mM KNO_3_	5 mM KNO_3_	50 mM KNO_3_
	Germination Inhibition in Dark Treatments (%)		
15/6	50.75	3.79	37.63
20/10	7.08	6.79	18.22
30/15	15.54	11.03	27.61
35/20	12.45	23.30	31.71

## Data Availability

All data are presented in this manuscript.
